# Mechanistic Insights into the Physiological and Meat Quality Responses of Broiler Chickens Fed Incremental Turmeric Rhizome Meal

**DOI:** 10.3390/ani15192849

**Published:** 2025-09-29

**Authors:** Uchenna Nonyelum Okonkwo, Christiaan Jacobus Smit, Chidozie Freedom Egbu

**Affiliations:** 1 Department of Agricultural Science, Faculty of Vocational and Technology Education, Alvan Ikoku Federal University of Education, Owerri P.M.B. 1033, Nigeria; okonkwoun@gmail.com; 2 Department of Agriculture and Animal Health, College of Agriculture and Environmental Sciences, University of South Africa, Florida Science Campus, Roodepoort 1709, South Africa; smitcj@unisa.ac.za

**Keywords:** antioxidant capacity, curcumin, growth performance, ileal digestibility, immunity, intestinal barrier function, intestinal morphology, meat quality, microbiome, nutrient transport markers

## Abstract

Natural products, such as turmeric rhizome meal (TRM), may hold the key to a sustainable solution to antimicrobial resistance rise and antibiotic prohibition in food-producing animals. This study evaluated the effects of dietary TRM at 0 (CON), 0.3 (TRM3), 0.6 (TRM6), and 0.9 g/kg (TRM9) on growth, nutrient digestibility, immunity, gut function, nutrient transport biomarkers, microbiome, and meat quality in 280 one-day-old male Ross 308 chicks over a 42-day feeding trial. Birds fed TRM indicated higher body weight gain and lower feed conversion ratio (*p* < 0.05). The TRM groups promoted higher (*p* = 0.001) serum immunoglobulin Y, immunoglobulin M, and interleukin-10 compared to the CON. Birds fed CON had higher interleukin-2 (*p* = 0.025), interleukin-6 (*p* = 0.027), and TNF-α (*p* = 0.008) levels compared to the TRM groups. Lactobacillus counts in jejunal villi and crypts were higher in the TRM groups than in the CON (*p* < 0.05). Dietary TRM increased electrogenic glucose and lysine transport, accompanied by up-regulation of claudin-5, zonula occludens 1, and mucin-2 expression (*p* < 0.05). In breast muscle, TRM fortification reduced malondialdehyde levels (*p* < 0.05) while increasing long-chain polyunsaturated fatty acids (*p* < 0.05). Thus, TRM is a potent, residue-free phytobiotic alternative to conventional antibiotic growth promoters in poultry systems.

## Simple Summary

Raising chickens without antibiotics is a major challenge for farmers, as antibiotics are often used to promote growth and prevent disease. However, indiscriminate antibiotic usage leads to the development of antibiotic-resistant bacteria. This study investigated whether adding turmeric root powder to chicken feed could be a safe and effective natural alternative. We fed varying small amounts of turmeric root powder to broiler chickens and measured its effects on their performance, physiology, and meat quality. We found that chickens fed turmeric root powder had better feed efficiency, stronger immune systems, and guts with more good bacteria. The meat from these chickens also had less fat damage and contained more beneficial nutrients. We conclude that supplementing turmeric root powder in poultry diets can help farmers raise healthier birds without antibiotics, ultimately providing consumers with a higher quality and safer meat.

## 1. Introduction

Future poultry production must strike a balance between bird health and productivity in the face of mounting concerns over antibiotic usage [1]. Historically, antibiotics have been administered in chicken feed as prophylaxis to attain these objectives; nevertheless, indiscriminate antibiotic usage has led to the advent of bacteria resistant to antibiotics [2], presenting considerable health concerns to humans and animals. In addressing this health concern, the National Agency for Food and Drug Administration and Control instituted a prohibition on the incorporation of antibiotic growth promoter (AGP) in animal feed in Nigeria (NAFDAC-RDAG-009-00). This regulation and other similar AGP prohibitions in the USA and European Union have prompted research into sustainable alternatives, such as probiotics [3], essential oils [4], prebiotics [5], and phytochemicals [6], to sustain the production efficiency and well-being of poultry.

Turmeric (*Curcuma longa*) contains proteins, fats, crude fibre, ascorbic acid, potassium, phosphorus, and polyphenols [7,8]. Polyphenols are a category of chemical molecules found in plants and examined for their crucial roles in disease therapy and as antioxidants [8]. These molecules are regarded as an environmentally friendly substitute for antibiotics in poultry production because there are no concerns for resistance and residues, and they are readily available from a variety of plant sources. These compounds have been demonstrated to be effective antibiotic replacements in enhancing animal efficiency, avoiding infection, and exhibiting anti-inflammatory, antibacterial, and antioxidant activity [9]. Curcumin, a polyphenol abundant in turmeric rhizome, has shown antiatherosclerosis, antiviral, antioxidant, lipid-modulating, antitumor, anticoagulant, antihepatic fibrosis, and anti-inflammatory effects in humans [10].

In poultry, researchers seeking antibiotic alternatives have noted that curcumin, owing to its anti-inflammatory, immunomodulatory, and antioxidant properties [11], positively influences production efficiency and product quality while sustaining bird health [8]. Additionally, turmeric rhizome fibre supports consistent bowel movements and nourishes beneficial gut bacteria by acting as a prebiotic [12], thereby promoting digestive health. Tannins, a different kind of polyphenol present in turmeric rhizome, are bitter and contribute to their antimicrobial properties [13].

The potential of these bioactive compounds to enhance barrier function and overall gut health is underscored by the biochemical mechanisms that underpin the effects of dietary polyphenols on gut microbiota and intestinal permeability [14,15]. Therefore, the intestinal mucus barrier function may be reinforced by the interaction of polyphenols with mucin, thereby improving gastrointestinal health [16]. This implies that dietary turmeric rhizome offers a sustainable means of sustaining poultry production efficiency and health. However, inconsistent data with turmeric rhizome supplementation in poultry feed [8,10,11] warrant a mechanistic insight.

For instance, 300 mg/kg turmeric rhizome extract in the diets of Wenchang chickens for 12 weeks increased their body weight gain (BWG) and feed efficiency [17]. In contrast, Ouedraogo et al. [18] and Sugiharto et al. [19] noted that dietary 4 g/kg turmeric powder and 1 g/kg acidified turmeric powder did not vary the feed intake, BWG, and feed efficiency of hybrid Dutch Blue chickens after 8 weeks and Lohmann broiler chickens after 35 days, respectively. Additionally, 4 g/kg TRM increased the nutrient digestibility coefficient for crude fibre, crude protein, and crude fat of Arbor Acre broiler chickens after 56 days [20]. Also, 30 mL/L turmeric oil administered in drinking water elevated the superoxide dismutase capacity of the breast meat of Arbor Acres broiler chickens after 21-day-frozen storage [21]. Consequently, this study examined the effects of incremental turmeric rhizome meal on the growth, apparent ileal digestibility, immunity, gut function, nutrient transport biomarkers, microbiome, and meat quality of broiler chickens.

## 2. Materials and Methods

### 2.1. Feed Ingredients Sourcing and Turmeric Rhizomes Preparation

The turmeric rhizomes were bought at Ose Okwe Odu Market (Onitsha, Anambra State, Nigeria). After being cleaned with tap water and cut using kitchen knives, they were allowed to dry at room temperature until their weight remained consistent. To create the needed turmeric rhizome meal (TRM), the rhizomes were ground to a size of 1 mm using a cutting mill (SM 100, Retsch GmbH, Haan, Germany). Additional feedstuffs were obtained from CEEKINGS Farm Feed Mill (Owerri, Imo State, Nigeria) for the experimental diet formulations.

### 2.2. Chemical Analysis of Turmeric Rhizome Meal and Experimental Diets

For 72 h, a 0.5 g TRM was continuously shaken while being macerated with 20 mL of methanol of high-performance liquid chromatography (HPLC) quality. A syringe filter with a pore size of 0.22 μm was used to filter the extracts. The TRM phenolic content was determined by HPLC (Agilent 1260 Infinity, Santa Clara, CA, USA) analysis using 20 μL of extract at a 1000 ppm concentration. A reverse-phase Nova-pack C18 column (9.9 mm × 150 mm; Waters, Milford, MA, USA) was used to accomplish the separation. Solvent A, which contained 0.1% trifluoroacetic acid (TFA) in 5% aqueous acetonitrile (ACN), and solvent B, which contained 0.1% TFA in ACN, comprised the mobile phase. The injection volume was maintained at 20 μL, the flow rate was set at 0.45 mL/min, and the column temperature was thermostatically maintained at 35 °C. Each sample’s total analysis duration was set at 25 min. A UV-VIS detector was used to detect HPLC chromatograms at 280 nm. The retention period and blasting with standards under identical conditions were used to identify each phenol (Table 1). The samples were subjected to HPLC analysis employing an isocratic solvent system consisting of 60 (A):40 (B) to estimate curcuminoids. At 425 nm, a UV-VIS detector was used to detect HPLC chromatograms.

The experimental diets (Table 2) were formulated to be isonitrogenous and isocaloric, meeting the nutritional needs of broiler chickens [22]. In these diets, TRM was added at amounts of 0 (CON), 0.3 (TRM3), 0.6 (TRM6), and 0.9 g/kg (TRM9) to a starter mash (1–14 days), grower pellet (15–28 days), and finisher pellet (29–42 days). To help determine the apparent ileal digestibility (AID) of nutrients, titanium dioxide (TiO_2_) was added as an inert marker to the finisher diets at a rate of 3 g/kg. According to the guidelines provided by the AOAC [23], the TRM and experimental diets were examined for dry matter (method 930.15; EcoTherm digital oven, LABOTEC, Midrand, South Africa), crude fat (method 920.39; ANKOM^XT−20^ Extractor, ANKOM Technology, Macedon, NY, USA), ash content (method 942.05; muffle furnace, Nabertherm, Lilienthal, Germany), and crude protein (method 976.05; Kjeldahl apparatus Büchi K-370, Flawil, Switzerland). An ANKOM^DELTA^ Fibre Analyser (ANKOM Technology, NY, USA) was used to measure crude fibre (CF) under Van Soest et al. [24] detergent procedures. A Gallenkamp ballistic bomb calorimeter was used to measure gross energy following Henken et al. [25]. Using a spectrometer (XRF Epsilon 4, Malvern Panalytical (Pty) Ltd., Randberg, South Africa), the amounts of calcium and phosphorus were ascertained. Following AOAC [23] guidelines, lysine and methionine were analysed using method 982.30 using an HPLC system that included a fluorescence detector.

### 2.3. Bird Husbandry and Study Design

Protocols involving birds were approved by the Ethics Committee of the Department of Animal Science and Fisheries, Imo State University, Owerri, Nigeria (IMSU/2022/033/A5) on 10 February 2022. One-day-old male Ross 308 chickens (n = 280 determined using G*Power 3.1.9.7 for Windows) were immunised against Newcastle and infectious bronchitis disease at GUFON Farms Enterprise in Owerri, Imo State, Nigeria. By body weight (BW), the birds were allotted randomly into the four dietary treatment groups. Each treatment group consisted of seven replicate pens (2.5 m length × 1.0 m width × 4.0 m height) as experimental units with wood shavings as litter. Each replicate pen contained 10 chicks, resulting in a stocking density of 4 chicks/m^2^ and a total of 70 chicks per treatment. Water and diet were given freely, while feed refusals were utilised to rectify data on feed intake. During the 42-day feeding trial, the bird house temperature was reduced step-by-step from 33 °C on day 1 to 23 °C by day 21 and maintained throughout the trial. The range of relative humidity was kept between 55 and 65%. The birds were exposed to a continuous lighting program (24L:0D) for the first 3 days to encourage initial feeding and water intake, followed by an 18 h light:6 h dark (18L:6D) schedule for the remainder of the trial.

### 2.4. Performance and Digestibility Measurement

For the starter, grower, and finisher phases, the mean BW and feed intake (FI) of the pen were noted. Consequently, FI, body weight gain (BWG), and feed conversion ratio (FCR) were computed for each period. Dead birds’ BW were noted when they happened and corrected in the FCR computations. Forty-two birds per group whose BW were nearer to their treatment mean BW were stunned and exsanguinated on day 42. For digestibility measurement, ileal digesta was taken from the lower half, two centimetres before the ileo-ceco-colic junction and between Meckel’s diverticulum of 14 carcasses per group and kept at −20 °C. The following formula was utilised to determine the AID of several nutrients:

(1) $$AID=100-\left.\left(\frac{\%\;marker\;in\;feed}{\%\;marker\;in\;ileum}\times \frac{\%\;nutrient\;in\;ileum}{\%\;nutrient\;in\;feed}\right.\right)$$

Furthermore, 21 carcasses per group were chosen to obtain the jejunum and cecum sections and were conserved in formalin for histological and gut microbiota analysis. For a later evaluation of lipid oxidation, amino acid, and fatty acid content, breast samples were collected, quickly frozen in liquid nitrogen, and then kept at −80 °C. Finally, the jejunum was taken from the remaining seven carcasses per group to identify the gene expression of nutrient transporters, tight junction proteins, cytokines, and Ussing chamber analysis.

### 2.5. Immunity Measurement

Blood samples were extracted from the vein in the wing of seven birds per group on day 42, which were moved right away into whole blood and serum tubes. The whole blood samples were used to determine blood immune cells (monocytes, lymphocytes, heterophils, heterophil/lymphocyte, and basophils) using a fully automated IDEXX^®^ LaserCyte Hematology Analyzer (IDEXX^®^ Laboratories Pty. S.A., Midrand, South Africa). The serum tube samples underwent centrifugation for 10 min at 3000 revolutions per minute, and the serum obtained was stored at −20 °C. Plasma levels of immunoglobulin A (IgA), immunoglobulin Y (IgY), immunoglobulin M (IgM), interleukin-2 (IL-2), interleukin-6 (IL-6), interleukin-10 (IL-10), and tumour necrosis factor-α (TNF-α) were quantified using an ELISA kit (Shanghai Enzyme Linked Biotechnology Co., Ltd., Shanghai, China) following the manufacturer’s guidelines. To begin, each well received 100 μL of plasma sample, which was then covered with the main antibody and stored at 37 °C for 15 min. In each well, after adding 100 μL of enzyme-conjugated secondary antibody solution, the mixture was allowed to sit at room temperature for two hours. Then, 100 μL of substrate solution was added and incubated for 30 min at room temperature. The enzyme process was stopped by applying a stop solution. The sample concentrations were calculated using a standard curve at an absorbance of 450 nm.

### 2.6. Intestinal Health and Functional Integrity

Following preservation in 4% phosphate-buffered formaldehyde, the gut tissue was dried and wrapped in paraffin. With a microtome (Type 1400 Fa. Leitz, Wetzlar, Germany), the histo-fixed specimens of the jejunum and cecum were divided into 5 μm slices. After drying at 37 °C, the tissue on the slide was dewaxed with xylene and rehydrated in graded ethanol. The jejunal and caecal tissues were stained using the Alcian blue pH 2.5 periodic acid-Schiff (AB-8GX, Sigma; Schiff reagents, Merck, Darmstadt, Germany) staining process [26]. A light microscope (Photomicroscope BX43F Olympus, Tokyo, Japan) fitted with a digital camera (Olympus DP72, Tokyo, Japan) was used to analyse images.

The cellSens imaging program (version 1.4, Olympus) was used to quantify crypt depth (CD) and villus length (VL). The number and location of goblet cells in the jejunal villi, crypts, and cecal crypts were determined automatically using QuPath software (v. 0.4.3). Each bird’s crypts and villi were measured ten times per segment. The villus-to-crypt ratio (V/C) was determined as VL/CD. The ratio of goblet cell count to area was measured independently for crypts and villi.

Samples of ileal and jejunal digesta were homogenised and centrifuged for 20 min at 4 °C at 5000× *g*. After that, the supernatants were transferred to Eppendorf tubes and centrifuged for 10 min at 4 °C at 12,000× *g*. According to Yalçin et al. [27], a viscometer (model DV-II + Pro, Brookfield Digital Viscometer, Brookfield Engineering Laboratories Inc., Stoughton, MA, USA) was used to test the viscosity of a 0.5 mL sample of the supernatant in centipoise (cP). For the microbiota analysis, only living bacterial species were included in the study since the caecal samples were processed to eliminate any dead cell DNA that could have been present. After that, the samples’ entire DNA was extracted. The bacterial community profiles of the samples were revealed by applying high-resolution melt analysis to the extracted DNA. To ascertain the total number of bacteria, *Lactobacillus* species, *Enterococcus* species, and *Escherichia coli* in the samples, qPCR was performed on the extracted DNA.

### 2.7. Gene Expression Measurement

For RNA extraction, 15 mg ±1.5 mg of each treatment’s jejunum tissue was used. Per the producer’s guidelines, total RNA was extracted using the NucleoSpin RNA Plus kit (Macherey–Nagel GmbH and Company KG, Düren, Germany). Next, the RNase-Free DNase Set kit (Qiagen, Hamburg, Germany) was used to purify the isolated RNA. The Bioanalyzer 2100 and the RNA 6000 Nano kit (Agilent Technologies, Waldbronn, Germany) were used to evaluate the RNA’s quality. In a SureCycler 8800 (Agilent Technologies), 500 ng of the purified RNA were reverse transcribed into cDNA using the SuperScript III Reverse Transcriptase kit (Thermo Fisher Scientific, Waltham, MA, USA). Tumour necrosis factor alpha (TNF-α), claudin 5, interleukin 8, mucin 2, and zonula occludens 1 (ZO-1) were among the genes whose expression levels were examined. β-actin, glyceraldehyde-3-phosphate dehydrogenase, and β2-microglobulin were employed as housekeeping genes for data normalisation (Table S1). Either a 1/25 or 1/50 dilution was employed for measurement to take into consideration variations in mRNA abundance among targets [28]. The AriaMax Real-Time PCR System (Agilent Technologies) was used to detect fluorescence quantitative polymerase chain reaction (RT-qPCR) in real time. For every RT-qPCR run, melting curves and PCR efficiency were used as standard quality criteria [26].

### 2.8. Ussing Chamber Analysis

Tissues from seven carcasses per treatment group were placed in three chambers for the electrophysiological measurements. The jejunum was put right away in ice-cold, oxygenated modified Krebs–Henseleit buffer with glucose in it after the stomach was cut open following euthanasia. The buffer contained glucose (10 mM glucose, 2 mM mannitol, 115 mM NaCl, 5 mM KCl, 25 mM NaHCO_3_, 0.6 mM NaH_2_PO_4_, 2.4 mM Na_2_HPO_4_, 1.2 mM MgCl_2_, 1.5 mM CaCl_2_, pH 7.4). A glass slide was used to remove the jejunum’s muscular layer, and the mucosal tissue was placed in the Ussing chamber with 0.79 cm^2^ of exposed space. To preserve tissue viability, Krebs–Henseleit buffer devoid of glucose was supplied to each chamber while being continuously oxygenated at 38 °C. Following tissue installation, a microcomputer-controlled voltage/current clamp (K. Mussler Scientific Instruments, Aachen, Germany) was used to detect the transepithelial potential difference to perform electrical experiments. To evaluate the tissue’s state, tissue conductance (Gt) was also measured. The reciprocal of tissue resistance is Gt, which is measured in millisiemens per square centimetre (mS⋅cm^2^). The voltage was adjusted to 0 mV to short-circuit the tissues after an equilibration time of around 20 to 30 min. Mannitol was concurrently administered to the serosal side of the epithelium to preserve osmotic balance while glucose (10 mM) was first supplied to the mucosal side to evaluate the absorption capacity. Once a steady baseline was achieved, lysine (10 mM) was added to the mucosal side as an extra substrate for the absorption test. Mannitol was then employed on the serosal side once more to maintain osmotic equilibrium. To promote the secretion of chloride ions, 0.1 mM carbachol solution was introduced to the basolateral compartment after the collecting time. Changes in tissue conductance (ΔGt) and short-circuit current (ΔIsc) were computed by comparing the average values throughout the last three minutes before and following each drug administration to depict the electrogenic transport mechanisms.

### 2.9. Meat Quality Assessment

In samples of breast meat, total antioxidant capacity (T-AOC), malonaldehyde dimethyl acetal (MDA), superoxide dismutase activity (SOD), and nitric oxide concentration were measured. In pursuance of the manufacturer’s instructions, this analysis was carried out using a commercial kit (ELISA Microplate Reader, Thermo Fisher Scientific, Waltham, MA, USA) produced by Nanjing Jiancheng Bioengineering Institute (Nanjing, China). Using a Hitachi amino acid analyser (L-8900), the sample’s amino acid content was ascertained following animal feeding stuffs-determination of amino acid content [29]. A 7890A gas chromatograph (Agilent, USA) was used to measure the amount of fatty acids in the sample following the meat and meat products-determination of total fat content [30].

### 2.10. Statistical Analysis

The normal option in the SAS 9.4 version [31] Proc Univariate statement was used to evaluate the normality of all the data that was gathered. ANOVA and the post hoc Tukey’s test were used to assess all data, except gene expression. The REST 2009 program was used to analyse gene expression [32]. Microbiota data were first transformed to logarithms and then examined similarly to the other data mentioned above. The statistical significance was set at *p* < 0.05 when evaluating differences between treatment groups for each parameter.

## 3. Results

### 3.1. Growth Performance

Table 3 shows that dietary incremental TRM promoted higher (*p* < 0.05) final BW and BWG during the starter, grower, and finisher phases, while the FCR was lower (*p* < 0.05) compared to the CON. Similarly, the overall performance showed that the TRM groups had comparable higher (*p* < 0.05) final BW and BWG while the FCR was lower (*p* < 0.05) compared to the CON.

### 3.2. Nutrients’ Apparent Ileal Digestibility

Table 4 shows that dietary incremental TRM had no difference between groups in the apparent ileal digestibility of 42-day-old broiler chickens (*p* > 0.05).

### 3.3. Immunity

The lymphocyte, immunoglobulin Y, immunoglobulin M, and interleukin-10 levels for broiler chickens fed TRM3, TRM6, and TRM9 were higher (*p* < 0.05) than for those fed CON (Table 5). The TRM group showed lower (*p* < 0.05) levels of heterophil/lymphocyte, interleukin-2, interleukin-6, and tumour necrosis factor-α compared to the CON.

### 3.4. Gut Morphology, Microbiota, and Function

For intestinal morphology (Table 6), the dietary TRM led to higher (*p* < 0.05) goblet cell counts at the villus compared to the CON. The intestinal structure of birds fed CON and TRM3 had similar lower (*p* < 0.05) goblet cell counts compared to TRM6 and TRM9 at the crypt. However, no difference between groups was noted for villus length, crypt depth, and villus length/crypt depth after TRM supplementation (*p* > 0.05). Total aerobic bacteria and *Lactobacillus* species were higher (*p* < 0.05) in the cecum of birds on TRM than in the CON. The cecum of birds fed TRM showed lower (*p* < 0.05) *Escherichia coli* compared to the CON. A reduction (*p* < 0.05) was noted in the viscosity of the ileum and jejunum digesta with dietary incremental TRM compared to the CON.

### 3.5. Immune and Barrier-Related Genes in the Jejunum Epithelium

Dietary incremental TRM had a significant effect on the relative expression ratio of claudin 5, mucin 2, and zonula occludens 1 (Table 7), with no differences between groups noted for tumour necrosis factor alpha and interleukin-8.

### 3.6. Intestinal Electrophysiological Function

Short-circuit current was higher (*p* < 0.05) for the TRM groups than the CON when glucose and lysine were added to the jejunum epithelium (Table 8). The Gt was lower (*p* < 0.05) in the TRM groups than in the CON when glucose was added to the jejunum epithelium.

### 3.7. Breast Meat Quality

Table 9 shows that breast meat samples from the CON had higher (*p* = 0.001) MDA than those from the TRM groups. The TRM groups indicated similar higher breast meat T-OAC (*p* = 0.005) and SOD (*p* = 0.001) capacity than those from the CON. Dietary TRM6 and TRM9 led to higher (*p* < 0.05) arginine, lysine, and phenylalanine content compared to CON and TRM3, which did not differ (*p* > 0.05). Diet TRM6 promoted the highest (*p* < 0.05) methionine and palmitic acid content, followed by TRM9 and TRM3, while the CON had the lowest values. The TRM groups had similar higher (*p* < 0.05) stearic acid, dihomo-gamma-linolenic acid, docosapentaenoic acid, and docosahexaenoic acid content in breast meat than the CON group. Diet TRM9 and TRM3 promoted the highest palmitoleic acid, followed by TRM6, and the lowest values were from the CON (*p* < 0.05).

## 4. Discussion

In recent years, there has been considerable interest in investigating phytochemicals as potential substitutes for antibiotics in poultry feed [33,34]. The necessity to reduce the buildup of antibiotic residues and antimicrobial resistance genes in poultry products is motivating this change [35]. Amid growing global restrictions on AGP, our study provides the first mechanistic evidence that TRM supports growth, modulates immunity and microbiome, fortifies gut barrier function, and enriches the meat quality of broiler chickens.

We observed that the final BW recorded in this study (1984–2110 g, Table 3) are lower than the standard performance objectives for the Ross 308 genotype. This was anticipated due to the metabolic cost of maintaining immune function and health in the absence of prophylactic agents and the variation in the nutrient composition and bioavailability of the locally sourced feedstuffs, which can modestly depress growth metrics. The observed increased BWG and decreased FCR in the present study are consistent with Wang, Huang, Zhou, Li, Zhou, Hou, Liu and Hu [17], who reported the same outcomes when 300 mg/kg turmeric rhizome extract was added to the diets of Wenchang chickens for 12 weeks. The reduced FCR in this study suggests that incremental TRM improved growth performance. This can be ascribed to the alteration of the inflammatory biomarkers and gut microbiota composition, improved goblet cell counts and barrier function, and upregulation of nutrient transport (Table 5, Table 6, Table 7 and Table 8). However, the similar FI observed in this study is at variance with Arslan et al. [36], who noted increased FI in Hubbard chickens after 35 days of adding 1.5 g/kg turmeric powder. Additionally, Ouedraogo, Nikiema, Sanou and Zoundi [18] and Sugiharto, Pratama, Yudiarti, Wahyuni, Widiastuti and Sartono [19] noted that dietary 4 g/kg turmeric powder and 1 g/kg acidified turmeric powder did not vary the FI, BWG, and FCR of hybrid Dutch Blue chickens after 8 weeks and Lohmann broiler chickens after 35 days, respectively. The noted variations in growth performance data among these studies can be attributed to differences in study duration and conditions, genotype, or the form and dosage of turmeric rhizome used.

Digestibility is a crucial nutritional indicator that gauges how well nutrients pass through the digestive system. Birds on CON showed comparable AID of nutrients measured in this study, suggesting that incremental TRM did not increase the AID of nutrients. Turmeric rhizome meal is rich in polyphenols such as curcumin (Table 1), which have been shown to stimulate endogenous digestive enzyme activity in chickens [37] and shrimps [38]. This upregulation of enzyme secretion can enhance the hydrolysis of macronutrients in the proximal gut, potentially increasing substrate availability for absorption in the ileum. However, curcumin and other polyphenols also exhibit binding properties that can form complexes with nutrients and digestive enzymes [39], thereby attenuating their activity in a dose-dependent manner. Such antagonistic effects may counterbalance the enzyme-stimulatory action, resulting in the comparable AID of nutrients with moderate dietary TRM levels. In contrast, 4 g/kg TRM increased the nutrient digestibility coefficient for crude fibre, crude protein, and crude fat of Arbor Acre broiler chickens after 56 days [20].

The immunological function of animals is primarily governed by the synergistic effect of immune cells, globulins, cytokines, and other components [40]. Dietary inclusion of TRM modulated innate and adaptive immune parameters in broiler chickens, as evidenced by elevated lymphocyte counts, IgY and IgM titres, and upregulated IL-10, alongside reductions in heterophil/lymphocyte, IL-2, IL-6, and TNF-α. The rise in lymphocyte counts suggests enhanced cellular immunity, while the boost in IgY and IgM reflects potentiation of humoral responses. Concurrently, the increase in IL-10 and the suppression of heterophil/lymphocyte, IL-2, IL-6, and TNF-α indicate a rebalancing of the cytokine milieu toward resolution of inflammation and immune homeostasis. Curcumin interferes with toll-like receptor 4-mediated signalling, thereby dampening downstream recruitment of myeloid differentiation main reaction gene 88 and activation of nuclear factor kappa B (NF-κB). By inhibiting the inhibitor of κB kinase activity, curcumin prevents the phosphorylation and subsequent proteasomal degradation of the inhibitor of κB alpha, thus retaining NF-κB in the cytoplasm and blunting transcription of IL-6 and TNF-α [41]. This mechanism explains the observed downregulation of IL-6 and TNF-α levels in TRM-fed birds. In agreement with our study, Huang, Wang, Zeng, Zang, Pan, Zhang, Yue, Wang, Zheng and Zhao [40] reported increased lymphocyte counts, IgY and IgM titres, and IL-10, alongside decreases in IL-2, IL-6, and TNF-α after 10 weeks in male Zi geese administered 200 mg/kg anthocyanins.

Dietary incremental TRM increased goblet cell counts in villi and crypts without altering villus height or crypt depth. This result is consistent with Ouyang et al. [42], who observed improved villi and crypt goblet cell counts with no variation in jejunum villus height or crypt depth when 1500 mg/kg white grape marc extract rich in polyphenols was administered to male Cobb 500 chickens after 35 days. Goblet cells secrete mucins that form the protective mucus layer, and their proliferation suggests that TRM polyphenols or fibre stimulated mucin biosynthesis through upregulation of mucin-2 expression as observed in this trial (Table 6 and Table 7). Similarly, elevated goblet cell counts and tightened junctional complexes through enhanced claudin and occludin gene expression have been reported with 1000 mg/kg dandelion supplementation for 42 days in male Arbor Acres chickens [43]. Mechanistically, curcumin can interact directly with mucin glycoproteins to stabilise the mucus gel and may activate the aryl hydrocarbon receptor–Nrf2 axis in enterocytes, promoting transcription of mucin genes and antioxidant defences [15,16]. The TRM’s polyphenols also modulated the caecal microbiota toward a more beneficial profile. Total aerobic counts and *Lactobacillus* species increased, while *Escherichia coli* declined significantly in TRM-fed broilers. Polyphenols can act as prebiotics, selectively inhibiting pathogenic bacteria and serving as substrates for beneficial microbes, which ferment them into short-chain fatty acids (SCFAs) that nourish enterocytes and reinforce barrier integrity. These microbial shifts possibly contributed to reduced digesta viscosity in the jejunum and ileum (Table 6), as lower viscosity enhances nutrient diffusion and limits mucin entrapment, further supporting nutrient absorption and barrier function. However, it is still not clear why these improvements were not translated into improved AID of nutrients in this study, warranting further examination.

In the present study, dietary incremental TRM elicited an upregulation of serum claudin 5, mucin 2, and zonula occludens-1 (ZO-1), while expression of TNF-α and IL-8 remained unchanged (Table 7). Polyphenols such as curcumin are known to inhibit NF-κB activation and modulate mitogen-activated protein kinase signalling, thereby relieving transcriptional repression of tight junction protein genes [10]. For example, dietary 400 mg/kg curcumin increased serum ZO-1 in Snowy White layers after 12 weeks [37]. Curcumin’s capacity to activate the Nrf2 pathway may further drive expression of tight junction components by augmenting cellular antioxidant defences and attenuating oxidative-stress–mediated barrier disruption [16].

Goblet cell-derived mucin is another critical arm of the gut’s physical barrier. In this trial, TRM increased mucin 2 expression, possibly via polyphenol-driven differentiation of goblet cells and upregulation of mucin 2 promoter activity via inhibition of pro-inflammatory signalling [15]. Comparable findings have been reported in Arian broiler Chickens fed 5 g/kg each of turmeric, thyme, and cinnamon for 42 days, where mucin 2 in the small intestine rose significantly [44], underscoring a shared mechanism among polyphenol-rich spices in enhancing the mucus layer. Interestingly, despite TRM’s barrier-enhancing effects, jejunal TNF-α and IL-8 gene expression did not differ from CON, suggesting that TRM’s immunomodulation may preferentially target upstream signalling nodes or post-transcriptional pathways rather than basal cytokine transcription. By contrast, 300 g/ton cinnamaldehyde (the principal bioactive in cinnamon) co-administered with 300 g/ton vitamin C significantly downregulated jejunal TNF-α mRNA while concomitantly upregulating ZO-1, occluden, and mucin 2 in 42-day-old female Arbor Acres broiler chickens [45]. This implies that depending on the plant and inherent cofactors, polyphenols can differentially modulate barrier and immune gene networks, reinforcing epithelial integrity.

The short-circuit current (ΔIsc) provides a direct readout of electrogenic nutrient transport across the jejunal epithelium. In our study, incremental dietary TRM raised the glucose-induced ΔIsc. This enhancement reflects greater Na^+^-coupled glucose uptake via sodium-dependent glucose cotransporter 1 (SGLT1) and likely also increased activity of glucose transporter 2 (GLUT2) on the basolateral membrane to ferry absorbed glucose into the circulation. Curcumin activates intracellular kinases that upregulate SGLT1 and GLUT2 expression in enterocytes [10], thereby accelerating glucose flux across the mucosa. A similar potentiation of ΔIsc was observed with 5 g/kg synbiotic (chicory-derived prebiotic plus *Enterococcus faecium*) supplementation in broilers for 21 days elevated jejunal ΔIsc by 333% versus 45% in CON [46]. Underscoring that enhancement of electrogenic nutrient transport is a common feature of gut-modulating feed additives. Moreover, white grape marc polyphenols have been shown to increase electrogenic ion currents in Cobb 500 chickens [42], which is consistent with observations for TRM-fed birds. The tissue conductance (Gt) inversely reflects tight junction integrity and paracellular leak. In this study, TRM lowered the glucose-stimulated ΔGt, indicating a tighter epithelial barrier. Curcumin interacts with mucin and tight junction proteins, promoting assembly of claudins and ZO-1 and reducing pore pathways between enterocytes [16]. Indeed, we observed upregulation of claudin 5 and ZO-1 in TRM-fed birds, which would be expected to curtail paracellular Na^+^ and water flux. White grape marc extracts likewise decreased jejunal ΔGt in Cobb 500 broilers [42], demonstrating that diverse polyphenol-rich additives converge on tightening the gut barrier. Overall, these Ussing chamber data provide functional evidence that TRM simultaneously improves nutrient absorptive capacity and fortifies intestinal barrier function in poultry.

Dietary incremental TRM improved breast meat antioxidant status, as evidenced by reduced MDA and elevated T-AOC and SOD activity. Curcumin acts as a free-radical scavenger, donating hydrogen atoms to lipid radicals [47], thereby interrupting the chain-propagation phase of lipid peroxidation. Curcumin’s diketone moiety chelates transition metal ions such as Fe^2+^ and Cu^2+^, further limiting hydroxyl radical formation via Fenton reaction [48]. In the sarcoplasmic reticulum of muscle fibres, this chelation curtails iron-catalysed breakdown of polyunsaturated fatty acids [49], reducing MDA formation and preserving membrane integrity. The up-regulation of endogenous antioxidant defences in TRM-fed birds, reflected by higher SOD activity, likely arises from curcumin’s modulation of the Nrf2 signalling pathway. Enhanced SOD converts superoxide anions into hydrogen peroxide, which is subsequently detoxified, maintaining redox balance within myocytes. Consistent with our study, 30 mL/L each of ginger and turmeric essential oil individually administered in drinking water elevated the SOD capacity of the breast meat of Arbor Acres broiler chickens after 21-day-frozen storage [21]. Similarly, Ross 308 broilers under cyclic heat stress from day 21–42 fed 1000 mg/kg ginger extract had increased T-AOC and decreased MDA capacity in breast muscle [50].

The amino acid composition in animal muscles alter the quality of meat, since it is crucial to defining the flavour of the meat. Dietary incremental TRM modulated breast meat amino acid composition, notably elevating arginine, lysine, methionine, and phenylalanine. Polyphenols enhance the expression and activity of nutrient-transporter genes in the intestinal epithelium through the activation of mTOR signalling and increased mucosal surface integrity [51]. In the present study, enhanced lysine-induced short-circuit currents in TRM-fed birds indicate upregulated electrogenic amino acid transport. By upregulation of ZO-1 and claudin-5, curcumin may further increase paracellular amino acid flux, ultimately boosting systemic availability and deposition into muscle fibres. Similar enhancements in muscle essential amino acids have been observed when Zi geese received 200 mg/kg anthocyanin [40].

The distinctive flavour of meat products is created via a sequence of chemical events involving fatty acids, including hydrolysis, thermal breakdown, oxidation, and the Maillard reaction, which result in the production of aldehydes, ketones, and alcohols [52]. Turmeric rhizome meal inclusion increased breast meat palmitic, stearic, palmitoleic, dihomo-γ-linolenic, docosapentaenoic, and docosahexaenoic acid content, likely reflecting TRM’s capacity to modulate hepatic and muscle lipid-metabolism enzymes [53]. Curcumin activates AMP-activated protein kinase, which in turn phosphorylates and inhibits acetyl-CoA carboxylase, reducing de novo lipogenesis, while simultaneously upregulating fatty-acid β-oxidation and desaturase gene expression via PPARα activation [54]. The net effect is a shift toward elongated and desaturated long-chain polyunsaturated fatty acids in muscle phospholipids, improving membrane fluidity and oxidative stability. Thus, the fatty acid remodelling observed with TRM reflects a mechanism by which dietary polyphenols orchestrate lipid-metabolic gene networks to enrich muscle PUFA content and quality.

## 5. Conclusions

Dietary 0.9 g/kg turmeric rhizome meal consistently enhanced broiler growth performance, strengthened intestinal barrier integrity (via up-regulation of claudin-5 and zonula occludens-1), optimised gut microbiota balance, and elevated nutrient transport capacity, which together translated into superior breast meat quality, characterised by increased essential amino acids and beneficial long-chain polyunsaturated fatty acids and robust antioxidant defences through direct radical scavenging and nuclear factor erythroid 2-mediated enzyme induction. However, 0.3 g/kg also significantly improved performance and health markers, potentially offering a cost-effective alternative for industry application. These multifunctional effects underscore the mechanistic synergy of turmeric rhizome meal polyphenols’ anti-inflammatory, prebiotic, and lipid-modulating actions, positioning turmeric rhizome meal as a sustainable, residue-free alternative to antibiotic growth promoters in modern poultry production. Despite these promising results, a limitation of this study is that the precise molecular pathways through which turmeric rhizome meal polyphenols exert their effects were not directly investigated. Future studies employing molecular techniques will be essential to delineate these specific mechanistic pathways and further validate the findings presented here.

## Figures and Tables

**Table 1:** Quantification (mg/kg dry matter) of the turmeric rhizome meal’s prevalent polyphenols and proximate composition (% dry matter).

Compound	Retention Time	Quantity
Gallic acid	5.06	5.47
Bis-demethoxycurcumin	9.07	9988
Demethoxycurcumin	10.02	19,700
Curcumin	11.06	24,700
Catechin	11.28	105.86
Caffeic Acid	12.15	121.48
t-Cinnamic acid	15.19	22.09
p-Coumaric acid	16.56	160.63
Ferrulic acid	18.17	468.34
Quercetin	19.74	2744
Salicylic acid	20.26	12.19
Proximate composition		
Dry matter		93.65
Crude fat		5.27
Crude protein		13.22
Ash		6.11
Crude fibre		8.37

The same HPLC procedure was also used to quantify the concentrations of bis-demethoxycurcumin, demethoxycurcumin, and curcumin in experimental diets.

**Table 2:** Ingredients (g/kg), proximate composition (% dry matter), essential amino acids (g/100 g dry matter), polyphenols (mg/kg), and gross energy of experimental diets.

	Starter (1–14 Days)				Grower (15–28 Days)				Finisher (29–42 Days)			
Ingredients	CON	TRM3	TRM6	TRM9	CON	TRM3	TRM6	TRM9	CON	TRM3	TRM6	TRM9
Turmeric rhizome meal	0.00	0.30	0.60	0.90	0.00	0.30	0.60	0.90	0.00	0.30	0.60	0.90
Corn	354	354	354	354	339	339	339	339	325	322	319	315
Soybean meal	334	334	334	334	296	296	296	296	273	273	274	276
Fish meal	34.6	37.3	37.9	40.0	30.0	29.7	29.4	29.1	27.1	25.8	32.5	38.5
Wheat offal	179	176	175	170	230	230	230	230	282	283	278	271
Palm kernel cake	50.0	50.0	50.0	52.6	60.0	60.0	60.0	60.0	47.0	50.0	50.0	52.7
Bone meal	15.1	15.1	15.0	15.0	14.0	14.0	14.0	14.0	12.5	12.5	12.5	12.5
Oyster shell	14.8	14.8	15.0	15.0	13.5	13.5	13.5	13.5	13.0	13.0	13.0	13.0
Common salt	3.00	3.00	3.00	3.00	2.00	2.00	2.00	2.00	1.50	1.50	1.50	1.50
Lysine	1.10	1.10	1.10	1.10	1.10	1.10	1.10	1.10	1.60	1.60	1.60	1.60
Methionine	2.40	2.40	2.40	2.40	2.40	2.40	2.40	2.40	2.30	2.30	2.30	2.30
^1^ Premix	12.0	12.0	12.0	12.0	12.0	12.0	12.0	12.0	12.0	12.0	12.0	12.0
Titanium dioxide	0.00	0.00	0.00	0.00	0.00	0.00	0.00	0.00	3.00	3.00	3.00	3.00
Total	1000	1000	1000	1000	1000	1000	1000	1000	1000	1000	1000	1000
Analysed composition												
Dry matter	90.1	90.3	90.4	90.3	90.5	90.4	90.0	90.4	89.8	89.7	90.1	89.9
Crude protein	22.4	22.6	22.2	22.2	20.2	20.5	20.8	21.0	18.2	18.5	18.8	18.0
Crude fibre	2.60	2.49	2.52	2.54	3.76	3.87	3.94	3.88	4.68	4.78	4.49	4.66
Crude fat	7.27	7.34	7.31	7.30	7.85	7.65	7.58	7.09	8.45	8.54	8.43	8.52
Ash	6.02	5.89	5.76	5.93	6.73	6.11	6.69	6.54	6.93	6.61	6.49	6.38
Calcium	1.04	1.03	1.02	1.02	1.14	1.13	1.12	1.12	0.87	0.82	0.85	0.81
Phosphorus	0.64	0.65	0.66	0.65	0.74	0.75	0.76	0.75	0.58	0.59	0.57	0.58
Lysine	0.70	0.71	0.72	0.71	0.69	0.73	0.68	0.71	0.82	0.85	0.84	0.86
Methionine	1.20	1.19	1.21	1.20	1.21	1.17	1.15	1.20	1.25	1.27	1.26	1.26
Bis-demethoxycurcumin	ND	3.30	6.00	9.10	ND	3.10	6.20	9.20	ND	3.20	6.10	9.00
Demethoxycurcumin	ND	5.90	12.0	17.7	ND	6.10	11.8	17.9	ND	6.00	11.9	18.0
Curcumin	ND	7.40	14.8	22.3	ND	7.50	14.9	22.2	ND	7.40	14.9	22.4
Gross energy (Kcal/kg)	3002	2999	2998	3005	3002	2995	3008	3005	3033	2911	2965	3010

CON = standard broiler chicken feed without turmeric rhizome meal; TRM3 = standard broiler chicken feed with 0.3 g/kg turmeric rhizome meal; TRM6 = standard broiler chicken feed with 0.6 g/kg turmeric rhizome meal; TRM9 = standard broiler chicken feed with 0.9 g/kg turmeric rhizome meal. ^1^ Premix: cyanocobalamin = 0.02 mg, folic acid = 1 mg, pantothenic acid = 10 mg, vitamin D3 = 2400 IU, vitamin K3 = 2 mg, nicotinic acid = 25 mg, vitamin B6 = 2 mg, vitamin A = 10,000 IU, vitamin B1 = 2 mg, vitamin E = 30 mg, vitamin B2 = 4 mg, manganese = 60 mg, selenium = 0.18 mg, copper = 8 mg, iron = 50 mg, iodine = 0.7 mg, and zinc = 40 mg. ND: not detected.

**Table 3:** Growth performance of broiler chickens fed dietary incremental turmeric rhizome meal for 42 days.

	^1^ Treatments					
	CON	TRM3	TRM6	TRM9	^2^ SEM	*p*-Value
Starter phase (1–14 days)						
Initial body weight (g)	40.76	40.80	40.75	40.78	0.14	0.863
Final body weight (g)	283.9 ^b^	289.6 ^a^	291.4 ^a^	293.9 ^a^	2.84	0.001
Body weight gain (g)	243.2 ^b^	248.8 ^a^	250.6 ^a^	253.1 ^a^	3.10	0.001
Feed intake (g/day)	370.3	370.8	373.1	375.0	4.00	0.601
Feed conversion ratio	1.523 ^a^	1.490 ^b^	1.489 ^b^	1.482 ^b^	0.01	0.001
Grower phase (15–28 days)						
Initial body weight (g)	283.9 ^b^	293.6 ^a^	291.4 ^a^	293.9 ^a^	2.84	0.001
Final body weight (g)	584.0 ^c^	604.4 ^b^	632.6 ^a^	632.2 ^a^	5.74	0.010
Body weight gain (g)	300.1 ^c^	310.8 ^b^	341.2 ^a^	338.3 ^a^	5.24	0.011
Feed intake (g/day)	496.7	494.8	540.8	534.5	6.44	0.462
Feed conversion ratio	1.655 ^a^	1.592 ^b^	1.585 ^b^	1.580 ^b^	0.02	0.001
Finisher phase (29–42 days)						
Initial body weight (g)	584.0 ^c^	604.4 ^b^	632.6 ^a^	632.2 ^a^	5.74	0.010
Final body weight (g)	1984	2065	2105	2110	25.8	0.004
Body weight gain (g)	1400 ^b^	1461 ^a^	1472 ^a^	1478 ^a^	15.5	0.009
Feed intake (g/day)	2621	2628	2608	2615	30.2	0.534
Feed conversion ratio	1.872 ^a^	1.799 ^b^	1.772 ^b^	1.769 ^b^	0.02	0.001
Overall (1–42 days)						
Initial body weight (g)	40.76	40.80	40.75	40.78	0.14	0.863
Final body weight (g)	1984 ^b^	2065 ^a^	2105 ^a^	2110 ^a^	25.8	0.004
Body weight gain (g)	1943 ^b^	2024 ^a^	2064 ^a^	2069 ^a^	24.6	0.008
Feed intake (g/day)	3488	3494	3522	3525	65.7	0.125
Feed conversion ratio	1.795 ^a^	1.726 ^b^	1.706 ^b^	1.703 ^b^	0.03	0.001

Means within a row with varying superscripts (a, b, c) vary significantly at *p* < 0.05. ^1^ Treatments: CON = standard broiler chicken feed without turmeric rhizome meal; TRM3 = standard broiler chicken feed with 0.3 g/kg turmeric rhizome meal; TRM6 = standard broiler chicken feed with 0.6 g/kg turmeric rhizome meal; TRM9 = standard broiler chicken feed with 0.9 g/kg turmeric rhizome meal. ^2^ SEM = standard error of the mean.

**Table 4:** Nutrient apparent ileal digestibility (%) of broiler chickens fed dietary incremental turmeric rhizome meal for 42 days.

	^1^ Treatments					
	CON	TRM3	TRM6	TRM9	^2^ SEM	*p*-Value
Crude protein	80.27	80.56	80.78	80.00	0.43	0.350
Crude fat	86.11	85.26	85.77	85.76	0.50	0.136
Crude fibre	67.32	65.75	63.65	62.00	0.67	0.631
Arginine	76.83	76.64	78.29	78.20	0.44	0.075
Histidine	71.27	72.17	74.42	72.83	0.35	0.085
Isoleucine	69.73	71.83	72.61	71.53	0.55	0.428
Leucine	71.14	71.62	72.36	72.84	0.46	0.741
Lysine	73.96	72.32	71.86	70.96	0.50	0.565
Methionine	76.34	75.22	75.31	75.58	0.45	0.786
Phenylalanine	71.77	72.45	73.54	74.22	0.58	0.222
Threonine	74.66	75.43	76.43	76.22	0.38	0.318
Valine	74.87	76.00	77.00	76.50	0.60	0.097
Alanine	68.00	68.50	70.00	70.00	0.45	0.653
Cystine	63.67	64.70	65.10	63.86	0.55	0.450
Glutamic acid	76.38	76.77	77.60	75.00	0.58	0.600
Glycine	67.00	67.00	68.50	65.90	0.44	0.272
Proline	73.00	72.50	70.20	70.10	0.33	0.545
Serine	69.20	69.77	67.80	68.90	0.66	0.290
Tyrosine	71.65	72.44	73.75	72.00	0.50	0.768
Calcium	41.53	42.77	39.92	45.45	0.70	0.665
Phosphorus	56.24	56.42	56.54	57.14	0.49	0.158
Potassium	80.19	81.27	80.37	82.40	0.47	0.316

^1^ Treatments: CON = standard broiler chicken feed without turmeric rhizome meal; TRM3 = standard broiler chicken feed with 0.3 g/kg turmeric rhizome meal; TRM6 = standard broiler chicken feed with 0.6 g/kg turmeric rhizome meal; TRM9 = standard broiler chicken feed with 0.9 g/kg turmeric rhizome meal. ^2^ SEM = standard error of the mean.

**Table 5:** Effect of dietary incremental turmeric rhizome meal on immune indicators of broiler chickens.

	^1^ Treatments					
	CON	TRM3	TRM6	TRM9	^2^ SEM	*p*-Value
Leukocyte counts (%)						
Lymphocytes	53.65 ^c^	63.57 ^b^	66.31 ^a^	66.40 ^a^	2.34	0.001
Heterophils	23.43	25.43	26.10	25.44	5.24	0.674
Heterophil/lymphocyte	0.44 ^a^	0.40 ^b^	0.39 ^b^	0.38 ^b^	0.01	0.002
Monocytes	4.67	4.56	4.76	4.77	4.21	0.437
Eosinophils	5.54	5.34	5.35	5.22	2.88	0.200
Basophils	2.00	3.00	1.58	3.00	3.54	0.706
Immunoglobulins (mg/mL)						
Immunoglobulin A	0.54	0.59	0.64	0.81	0.05	0.513
Immunoglobulin Y	3.17 ^b^	3.74 ^a^	3.79 ^a^	3.75 ^a^	0.11	0.001
Immunoglobulin M	0.33 ^c^	0.40 ^b^	0.50 ^a^	0.50 ^a^	0.02	0.001
Cytokines (pg/mL)						
Interleukin-2	48.45 ^a^	47.62 ^b^	46.04 ^c^	46.05 ^c^	2.06	0.025
Interleukin-6	34.18 ^a^	34.24 ^a^	30.44 ^b^	30.87 ^b^	1.50	0.027
Interleukin-10	10.02 ^b^	12.25 ^a^	12.29 ^a^	12.29 ^a^	2.25	0.001
Tumour necrosis factor-α	48.48 ^a^	44.71 ^b^	42.94 ^b^	44.21 ^b^	4.00	0.008

Means within a row with varying superscripts (a, b, c) vary significantly at *p* < 0.05. ^1^ Treatments: CON = standard broiler chicken feed without turmeric rhizome meal; TRM3 = standard broiler chicken feed with 0.3 g/kg turmeric rhizome meal; TRM6 = standard broiler chicken feed with 0.6 g/kg turmeric rhizome meal; TRM9 = standard broiler chicken feed with 0.9 g/kg turmeric rhizome meal. ^2^ SEM = standard error of the mean.

**Table 6:** Effect of dietary incremental turmeric rhizome meal on intestinal morphology, goblet cell count, gut microbiota, and intestinal viscosity of broiler chickens.

	^1^ Treatments					
	CON	TRM3	TRM6	TRM9	^2^ SEM	*p*-Value
Jejunum						
Villus length (μm)	1637	1688	1649	1711	25.2	0.188
Crypt depth (μm)	232	233	233	244	5.44	0.260
Villus length /crypt depth	7.06	7.24	7.08	7.01	0.30	0.870
Villus goblet cells (×10^3^/mm^2^)	0.87 ^b^	1.19 ^a^	1.20 ^a^	1.21 ^a^	0.05	0.001
Crypt goblet cells (×10^3^/mm^2^)	2.65 ^b^	2.85 ^b^	3.90 ^a^	4.03 ^a^	0.10	0.001
Cecum						
Crypt depth (μm)	335	337	339	340	4.65	0.794
Crypt goblet cells (×10^3^/mm^2^)	0.66	0.64	0.74	0.76	0.03	0.148
Cecal microorganisms (log_10_cfu/g)						
Total aerobic bacteria	6.87 ^b^	7.21 ^a^	7.25 ^a^	7.27 ^a^	0.09	0.009
*Lactobacillus* species	4.49 ^b^	5.38 ^a^	5.59 ^a^	5.32 ^a^	0.30	0.001
*Enterococcus* species	4.89	5.33	5.28	5.39	0.18	0.109
*Escherichia coli*	4.99 ^a^	4.33 ^b^	4.27 ^b^	4.29 ^b^	0.13	0.004
Viscosity (cP)						
Jejunum	2.42 ^a^	1.85 ^b^	1.80 ^b^	1.72 ^b^	0.08	0.001
Ileum	2.92 ^a^	2.44 ^b^	2.54 ^b^	2.50 ^b^	0.09	0.001

Means within a row with varying superscripts (a, b) vary significantly at *p* < 0.05. ^1^ Treatments: CON = standard broiler chicken feed without turmeric rhizome meal; TRM3 = standard broiler chicken feed with 0.3 g/kg turmeric rhizome meal; TRM6 = standard broiler chicken feed with 0.6 g/kg turmeric rhizome meal; TRM9 = standard broiler chicken feed with 0.9 g/kg turmeric rhizome meal. ^2^ SEM = standard error of the mean.

**Table 7:** Effect of dietary incremental turmeric rhizome meal on relative expression ratio of immune response and barrier function in the jejunum of 42-day-old broiler chickens.

	CON vs. TRM3		CON vs. TRM6		CON vs. TRM9		TRM3 vs. TRM6		TRM3 vs. TRM9		TRM6 vs. TRM9	
	Ratio	*p*-Value	Ratio	*p*-Value	Ratio	*p*-Value	Ratio	*p*-Value	Ratio	*p*-Value	Ratio	*p*-Value
^1^ TNF-α	1.50	0.548	1.15	0.354	1.09	0.739	0.79	0.639	0.62	0.329	0.79	0.415
Claudin 5	1.06	0.034	1.19	0.030	1.35	0.001	0.92	0.296	0.89	0.273	0.90	0.766
Interleukin-8	0.68	0.373	0.69	0.405	0.70	0.297	0.65	0.529	0.70	0.876	0.77	0.385
Mucin 2	0.95	0.006	1.02	0.007	1.23	0.023	1.34	0.230	0.92	0.283	1.29	0.271
^2^ ZO-1	1.00	0.005	1.20	0.026	1.30	0.040	0.89	0.608	0.79	0.864	0.89	0.527

CON = standard broiler chicken feed without turmeric rhizome meal; TRM3 = standard broiler chicken feed with 0.3 g/kg turmeric rhizome meal; TRM6 = standard broiler chicken feed with 0.6 g/kg turmeric rhizome meal; TRM9 = standard broiler chicken feed with 0.9 g/kg turmeric rhizome meal. ^1^ TNF-α = tumour necrosis factor alpha; ^2^ ZO-1 = zonula occludens 1.

**Table 8:** Effect of dietary incremental turmeric rhizome meal on short-circuit current and tissue conductance in the jejunum epithelium of 42-day-old broiler chickens.

	^1^ Treatments					
	CON	TRM3	TRM6	TRM9	^2^ SEM	*p*-Value
^3^ |ΔIsc| (μA/cm^2^)						
Glucose	4.30 ^b^	6.86 ^a^	7.70 ^a^	8.85 ^a^	1.05	0.001
Lysine	0.65 ^b^	0.78 ^ab^	0.89 ^a^	0.98 ^a^	0.10	0.001
Carbachol	2.46	2.00	3.01	2.98	0.45	0.108
^4^ |ΔGt| (mS/cm^2^)						
Glucose	0.24 ^a^	0.13 ^b^	0.09 ^b^	0.11 ^b^	0.02	0.010
Lysine	0.30	0.25	0.20	0.17	0.03	0.155
Carbachol	0.55	0.25	0.44	0.35	0.12	0.756

Means within a row with varying superscripts (a, b) vary significantly at *p* < 0.05. ^1^ Treatments: CON = standard broiler chicken feed without turmeric rhizome meal; TRM3 = standard broiler chicken feed with 0.3 g/kg turmeric rhizome meal; TRM6 = standard broiler chicken feed with 0.6 g/kg turmeric rhizome meal; TRM9 = standard broiler chicken feed with 0.9 g/kg turmeric rhizome meal. ^2^ SEM = standard error of the mean; ^3^ |ΔIsc| = change in short-circuit current; ^4^ |ΔGt| = change in tissue conductance.

**Table 9:** Effect of dietary incremental turmeric rhizome meal on breast meat antioxidant capacity, amino acid (%), and fatty acid (%) composition.

	^1^ Treatments					
	CON	TRM3	TRM6	TRM9	^2^ SEM	*p*-Value
Antioxidant capacity						
Malondialdehyde (nmol/g)	0.765 ^a^	0.704 ^b^	0.675 ^b^	0.657 ^b^	0.30	0.001
Total antioxidant capacity (U/mL)	4.54 ^b^	6.67 ^a^	6.11 ^a^	6.89 ^a^	1.00	0.005
Superoxide dismutase (U/mL)	130 ^b^	149 ^a^	147 ^a^	145 ^a^	4.99	0.001
Nitric oxide (μmol/mL)	16.5	14.5	13.7	15.4	6.54	0.675
Essential amino acid composition						
Arginine	5.01 ^b^	5.01 ^b^	5.53 ^a^	5.56 ^a^	0.22	0.032
Histidine	3.56	4.12	3.77	3.56	0.46	0.718
Isoleucine	4.36	4.06	3.99	4.03	0.34	0.405
leucine	5.53	6.00	5.77	5.87	0.76	0.876
Lysine	7.16 ^b^	7.20 ^b^	7.78 ^a^	7.75 ^a^	0.25	0.015
Methionine	3.03 ^c^	3.22 ^b^	3.47 ^a^	3.25 ^b^	0.10	0.027
Phenylalanine	4.90 ^b^	4.90 ^b^	5.46 ^a^	5.40 ^a^	0.12	0.003
Threonine	3.60	3.45	3.35	3.50	0.45	0.642
Valine	4.37	4.32	4.30	4.23	0.65	0.378
Fatty acid composition						
Pentadecanoic acid	0.10	0.09	0.10	0.09	0.01	0.698
Palmitic acid	26.8 ^c^	28.1 ^b^	29.6 ^a^	28.4 ^b^	0.50	0.029
Stearic acid	7.18 ^b^	7.67 ^a^	7.67 ^a^	7.66 ^a^	0.26	0.007
Arachidic acid	0.08	0.07	0.07	0.07	0.01	0.775
Palmitoleic acid	3.49 ^c^	4.08 ^a^	3.80 ^b^	4.15 ^a^	0.05	0.021
Oleic acid	36.8	36.5	37.2	37.0	2.45	0.650
Dihomo-gamma-linolenic acid	0.06 ^b^	0.09 ^a^	0.11 ^a^	0.11 ^a^	0.01	0.001
Docosapentaenoic acid	0.60 ^b^	1.02 ^a^	1.00 ^a^	1.01 ^a^	0.01	0.040
Docosahexaenoic acid	0.06 ^b^	0.10 ^a^	0.10 ^a^	0.10 ^a^	0.01	0.002
Eicosadienoic acid	0.13	0.13	0.11	0.12	0.01	0.076

Means within a row with varying superscripts (a, b, c) vary significantly at *p* < 0.05. ^1^ Treatments: CON = standard broiler chicken feed without turmeric rhizome meal; TRM3 = standard broiler chicken feed with 0.3 g/kg turmeric rhizome meal; TRM6 = standard broiler chicken feed with 0.6 g/kg turmeric rhizome meal; TRM9 = standard broiler chicken feed with 0.9 g/kg turmeric rhizome meal. ^2^ SEM = standard error of the mean.

## Data Availability

The original contributions presented in this study are included in the article/Supplementary Materials. Further inquiries can be directed to the corresponding author.

## Supplementary Materials

The following supporting information can be downloaded at: https://www.mdpi.com/article/10.3390/ani15192849/s1, Table S1. Primer sequences used for quantitative real-time polymerase chain reaction analysis.

## Abbreviations

The following abbreviations are used in this manuscript:

AID	Apparent ileal digestibility
BW	Body weight
BWG	Body weight gain
FCR	Feed conversion ratio
FI	Feed intake
GLUT2	Glucose transporter 2
Gt	Tissue conductance
IL-2	Interleukin-2
IL-6	Interleukin-6
IL-8	Interleukin-8
IL-10	Interleukin-10
Isc	Short-circuit current
IgA	Immunoglobulin A
IgM	Immunoglobulin M
IgY	Immunoglobulin Y
MDA	Malondialdehyde
mTOR	Mechanistic target of rapamycin
NF-κB	Nuclear factor kappa B
Nrf2	Nuclear factor erythroid 2
PPARα	Peroxisome proliferator-activated receptor alpha
PUFA	Polyunsaturated fatty acids
SCFAs	Short-chain fatty acids
SGLT1	Sodium-dependent glucose cotransporter 1
SOD	Superoxide dismutase
T-AOC	Total antioxidant capacity
TNF-α	Tumour necrosis factor alpha
TRM	Turmeric rhizome meal
ZO-1	Zonula occludens-1
