# Supplementary material for: Mechanistic Insights into the Physiological and Meat Quality Responses of Broiler Chickens Fed Incremental Turmeric Rhizome Meal
Source: Animals (Basel). 2025 Sep 29;15(19):2849. doi: 10.3390/ani15192849 (PMC12524245; doi:10.3390/ani15192849)
Supplement: Supplementary file 1 [file animals-15-02849-s001.zip › animals-3886119-supplementary.pdf]

Table S1. Primer sequences used for quantitative real-time polymerase chain reaction analysis.

Gene	Forward (5' → 3')	Reverse (5' → 3')	Amplicon size	Accession number
Tumour necrosis factor alpha	GCCCTTCCTGTAACCAGATG	ACACGACAGCCAAGTCAACG	71	NM_204267.1
Claudin 5	AGGTGTCAGCCTTCATCGAC	CCAGGATGGAATCGTACACC	123	NM_204201
Interleukin 8	GGCTTGCTAGGGGAAATGA	AGCTGACTCTGACTAGGAAACTGT	136	AJ009800
Mucin 2	ATTGAAGCCAGCAATGGTGT	TTGTTGGCCTTGTCATCAAA	125	ENSGALG00000006744CGNC
Zonula occludens 1	AAGTGGGAAGAATGCCAAAA	GGTCCTTGGATCCCGTATCT	133	ENSGALG00000003970CGNC
Housekeeping Genes				
β-actin	GTGGATCAGCAAGCAGGAGT	ATCCTGAGTCAAGCGCCAAA	182	NM_205518.2
Glyceraldehyde-3-phosphate dehydrogenase	ACATGGCATCCAAGGAGTGAG	GGGGAGACAGAAGGGAACAGA	144	NM_204305.1
β2-microglobulin	AAGGAGCCGCAGGTCTAC	CTTGCTCTTTGCCGTCATAC	150	Z48921.1
